# Opportunities and Challenges of Chatbots in Ophthalmology: A Narrative Review

**DOI:** 10.3390/jpm14121165

**Published:** 2024-12-21

**Authors:** Mehmet Cem Sabaner, Rodrigo Anguita, Fares Antaki, Michael Balas, Lars Christian Boberg-Ans, Lorenzo Ferro Desideri, Jakob Grauslund, Michael Stormly Hansen, Oliver Niels Klefter, Ivan Potapenko, Marie Louise Roed Rasmussen, Yousif Subhi

**Affiliations:** 1 Department of Ophthalmology, Kastamonu University, Training and Research Hospital, 37150 Kastamonu, Türkiye; mcsabaner@kastamonu.edu.tr; 2 Department of Ophthalmology, Inselspital, University Hospital Bern, University of Bern, 3010 Bern, Switzerland; r.anguitahenriquez@nhs.net (R.A.); lorenzo.ferrodesideri@insel.ch (L.F.D.); 3 Moorfields Eye Hospital National Health Service Foundation Trust, London EC1V 2PD, UK; 4 The CHUM School of Artificial Intelligence in Healthcare, Montreal, QC H2X 0A9, Canada; 5 Cole Eye Institute, Cleveland Clinic, Cleveland, OH 44195, USA; 6 Department of Ophthalmology & Vision Sciences, University of Toronto, Toronto, ON M5T 2S8, Canada; michael.balas@mail.utoronto.ca; 7 Department of Ophthalmology, Innlandet Hospital Trust, 2382 Brumendal, Norway; lars.christian.boberg-ans@rsyd.dk; 8 Graduate School for Health Sciences, University of Bern, 3012 Bern, Switzerland; 9 Department of Ophthalmology, Odense University Hospital, 5000 Odense, Denmark; 10 Department of Clinical Research, University of Southern Denmark, 5230 Odense, Denmark; 11 Department of Ophthalmology, Vestfold Hospital Trust, 3103 Tønsberg, Norway; 12 Department of Ophthalmology, Rigshospitalet, 2100 Copenhagen, Denmark; michael.stormly.hansen@regionh.dk (M.S.H.); oliver.niels.klefter.01@regionh.dk (O.N.K.); marie.louise.roed.rasmussen@regionh.dk (M.L.R.R.); 13 Department of Clinical Medicine, University of Copenhagen, 1172 Copenhagen, Denmark

**Keywords:** artificial intelligence, Bard, Bing, ChatGPT, Claude, e-learning, Gemini, large language model, ophthalmology

## Abstract

Artificial intelligence (AI) is becoming increasingly influential in ophthalmology, particularly through advancements in machine learning, deep learning, robotics, neural networks, and natural language processing (NLP). Among these, NLP-based chatbots are the most readily accessible and are driven by AI-based large language models (LLMs). These chatbots have facilitated new research avenues and have gained traction in both clinical and surgical applications in ophthalmology. They are also increasingly being utilized in studies on ophthalmology-related exams, particularly those containing multiple-choice questions (MCQs). This narrative review evaluates both the opportunities and the challenges of integrating chatbots into ophthalmology research, with separate assessments of studies involving open- and close-ended questions. While chatbots have demonstrated sufficient accuracy in handling MCQ-based studies, supporting their use in education, additional exam security measures are necessary. The research on open-ended question responses suggests that AI-based LLM chatbots could be applied across nearly all areas of ophthalmology. They have shown promise for addressing patient inquiries, offering medical advice, patient education, supporting triage, facilitating diagnosis and differential diagnosis, and aiding in surgical planning. However, the ethical implications, confidentiality concerns, physician liability, and issues surrounding patient privacy remain pressing challenges. Although AI has demonstrated significant promise in clinical patient care, it is currently most effective as a supportive tool rather than as a replacement for human physicians.

## 1. Introduction

Chatbots are among the most prominent artificial intelligence (AI) tools of the “AI age” and have revolutionized how users interact with technology over the past decade [1,2]. These tools leverage conversational interfaces to provide easy access to information and perform a range of tasks, such as text generation, language understanding, question answering, translation, and summarization [3,4,5,6]. A chatbot is an automated software application designed to simulate and facilitate conversations with human users, typically through text- or voice-based interactions. Modern chatbots often utilize large language models (LLMs), which are deep learning systems trained on massive datasets to understand a wide range of linguistic patterns [7,8]. By applying advanced natural language processing (NLP) techniques, LLM-based chatbots can deliver accurate, contextually relevant responses in multiple languages. AI-based LLM chatbots have recently garnered substantial attention for their ability to generate human-like responses in interactive dialogue formats, as well as for their accessibility, user-friendly interfaces, and minimal learning curve [9,10,11]. Their versatility has led to their widespread adoption across diverse fields, including e-learning, personalized medicine, customer service, autonomous vehicles, data analysis, and software development [1,2,3,4,5,6].

The focus on chatbots in ophthalmology is driven by their potential to address several unique challenges and create significant opportunities within the field. Ophthalmology is a highly specialized domain that often involves a substantial patient load, requiring efficient communication and management systems to ensure timely diagnosis and treatment [12,13]. Also, due to various demographic challenges, a shortage of ophthalmologists, high patient volumes, and the burdens on this specialty, the development and integration of chatbots into ophthalmology have become essential. Over the coming decades, the number of persons with visual impairment is expected to rise significantly [14,15]. Demographic changes are likely to result in an increasing number of patients with chronic, age-related conditions, such as age-related macular degeneration and glaucoma, which will necessitate a greater capacity for long-term monitoring [16,17]. Although the number of ophthalmologists appears to be increasing worldwide, it seems likely that new technological solutions will have to be adopted to meet these future challenges [18]. Chatbots offer scalable solutions by automating patient interactions, answering frequently asked questions, and assisting with appointment scheduling, thereby alleviating the administrative burdens on healthcare providers [19]. Moreover, ophthalmology involves a wide range of conditions, many of which require patient education about disease progression, treatment adherence, and preventive measures. Chatbots provide a cost-effective, accessible, and user-friendly medium for delivering personalized education and support, even in remote or underserved areas [19]. For example, chatbots can guide patients on pre- and post-surgical care or provide reminders about routine eye examinations, which are critical for the early detection of diseases, such as glaucoma or diabetic retinopathy. In addition to addressing these challenges, chatbots also create opportunities for data collection and analysis. By interacting with patients, chatbots can gather valuable insights into symptoms, treatment outcomes, and patient satisfaction. This information can be used to enhance clinical decision-making, tailor interventions, and improve the overall quality of care. Furthermore, the integration of chatbots into ophthalmology-focused APIs or electronic health record (EHR) systems can streamline workflows, enabling healthcare professionals to focus more on complex clinical tasks [20]. Ultimately, the use of chatbots in ophthalmology aligns with the broader trend of digital health innovation, offering a pathway to enhance efficiency, accessibility, and patient engagement in a rapidly evolving healthcare landscape. By addressing the existing challenges and leveraging emerging opportunities, chatbots have the potential to significantly transform the delivery of ophthalmic care. AI solutions in general, and chatbots in particular, have shown promising results in recent years and could become useful tools [9].

The research comparing human performance to that of chatbots on multiple-choice question (MCQ) assessments has gained significant attention and has continued to evolve [21,22,23,24]. Chatbots have successfully passed various medical licensing exams, and their performance on different medical specialty board exams has also been noteworthy [25,26,27,28,29,30,31,32,33]. These successes have naturally raised the question of whether chatbots could eventually function as artificial physicians—or even artificial ophthalmologists. Following their demonstrated proficiency in handling MCQs, the next challenge for chatbots has been case management and providing recommendations from a scientific and specialized ophthalmological perspective [34,35,36,37,38,39,40,41,42,43].

Initially, discussions focused on validating the appropriateness of chatbots’ responses to specific patient questions. However, the current research has shifted to understanding how chatbots could offer case recommendations from a physician’s viewpoint. While the early iterations of chatbots struggled to provide reliable answers for clinical case management and treatment recommendations, advancements in AI have enabled them to deliver more accurate and scientifically grounded suggestions. Despite these improvements, the inherent fallibility of human decision-making does not automatically justify reliance on chatbots.

Hence, in this narrative review, we focus on studies evaluating chatbots’ responses to open-ended and closed-ended ophthalmological queries, highlighting the opportunities and challenges associated with their integration into clinical practice. This narrative review examines ophthalmological studies involving chatbots, with a primary focus on the widely adopted standalone chatbot platforms, while also considering systems developed using application programming interfaces (API).

### 1.1. AI-Based LLM Chatbots and Related API Tools

In today’s era of technology and AI, it is increasingly essential for ophthalmologists to be knowledgeable about chatbots, as both healthy individuals and patients frequently turn to AI, in addition to internet searches, to seek medical information and advice. There are numerous models and versions of AI-based LLM chatbots and related API tools in this rapidly evolving field, with some of the most studied being ChatGPT, Gemini (formerly Bard), Copilot, Claude, LLaMA, and Bing, among others (Table 1).

#### 1.1.1. ChatGPT

ChatGPT, developed by OpenAI, is one of the most widely used and advanced LLMs available today. Initially launched in November 2022 with the GPT-3.5 model, ChatGPT quickly gained traction across academia, professional services, education, and general information access, thanks to its capabilities in generating coherent and contextually relevant responses [44,45].

In early 2023, ChatGPT saw significant improvements with the introduction of the GPT-4 model, which had enhanced abilities for handling complex inquiries; improved contextual understanding; and more accurate, reliable information delivery. This advanced version of ChatGPT has benefited from OpenAI’s ongoing research and development, as well as regular updates informed by extensive user feedback [46,47].

Notably, ChatGPT is frequently used in academic research and other knowledge-intensive fields due to its ability to provide quick access to information and support complex queries. It is also available via OpenAI’s API services, enabling developers to integrate ChatGPT into various applications and websites. By 2023, ChatGPT’s broad functionality and integration capabilities made it a popular tool across a diverse user base [46,47,48].

#### 1.1.2. Gemini

Gemini, formerly known as Bard, is one of the latest AI-based LLMs developed by Google. Initially launched as Bard in early 2023, it represented Google’s entrance into the LLM space as a competitor to OpenAI’s ChatGPT [29,49,50]. Bard was initially available only in the United States and the United Kingdom. Over the following few months, Bard underwent continuous updates, with improvements in its response quality and additional functionality.

In late 2023, Google rebranded Bard as Gemini, signaling a shift toward more advanced, versatile AI features designed to integrate more closely with Google’s suite of services. The launch of Gemini was seen as an advancement, offering improved contextual understanding, faster response times, and better integration with Google’s search engine and data ecosystem [49,51].

Gemini has since become widely accessible across multiple regions and supports various applications, from educational assistance to professional tasks in fields like healthcare, research, and customer service. It also provides integration with other Google tools. With continuous improvements and expansions, Gemini has become a prominent tool in the LLM landscape [49].

#### 1.1.3. Other Chatbots

Microsoft’s Copilot, formerly Bing Chat, was introduced in 2021 as an AI-powered assistant integrated into its Microsoft™ Office and 365 applications [52,53]. Designed to streamline productivity tasks, Copilot assists users with document generation, summarization, and interactive support within Microsoft™ applications. By 2023, Copilot was enhanced with advanced NLP abilities, integrating OpenAI’s GPT-4 capabilities to improve task accuracy and deliver a more seamless user experience.

Perplexity AI, launched in 2022, operates as a search-based chatbot that combines NLP with a knowledge graph to provide accurate, reference-linked responses to user queries [54,55]. Unlike other models, Perplexity’s focus lies in creating more contextually aware, citation-backed answers, which has made it popular for research purposes and fact-based inquiries. The continuous updates of the model have increased its accuracy and reliability, particularly in academic and technical fields [54,55].

Aeyeconsult, launched in 2020, was developed specifically for ophthalmology with the goal of facilitating ophthalmic diagnostics through AI. This tool combines advanced image analysis with AI-guided consultations to assist in areas like diabetic retinopathy screening. By 2023, Aeyeconsult had integrated more sophisticated image recognition capabilities, enabling ophthalmologists to access quicker, more accurate diagnostics, thus supporting both medical training and clinical decision-making [56].

Claude, developed by Anthropic in 2023, emerged as an AI assistant built on the principles of “Constitutional AI”, emphasizing safe and ethical use. Claude was designed to produce controlled and reliable responses, offering an alternative to other LLMs [54,57]. Claude’s capabilities were further refined to enhance its contextual understanding and ethical safety, positioning it as a model that prioritizes responsible AI interaction.

Cohere was founded in 2019 by former Google Brain researchers, aiming to make large language models accessible for business applications, particularly for text generation and language understanding. By 2022, Cohere introduced its Command R+ model, optimized for retrieval-augmented generation applications [58]. This update catered specifically to controlled and accurate text output for commercial contexts and business-specific LLM applications.

Falcon, a product of the Technology Innovation Institute, was released as an open-source model in 2023 and has been recognized for its efficiency and high-quality outputs [59]. Its design focused on enhancing computational efficiency and language capability for diverse industry applications. In 2024, Falcon received a substantial update to Falcon 180B, positioning it as a tool for multilingual research and practical applications.

Grok, developed by xAI in 2023, was created as an advanced conversational AI designed for enterprise use [60]. The release of Grok-2 in August 2024 introduced multimodal functionality. Integrated with the X platform, Grok-2 improved real-time engagement and expanded its applications, establishing itself as a versatile solution in the growing field of AI technologies.

LLaMa, released by Meta AI in February 2023, targeted the academic and scientific community, with an emphasis on efficient language processing for research applications. This model was developed to be accessible and adaptable for scientific inquiry and advanced language applications [61,62]. A key distinguishing feature of LLaMa is its open-access policy, which enables researchers, educators, and developers to freely utilize and modify the model for their specific needs, fostering innovation and collaboration in AI research. In July 2023, Meta updated the model to LLaMa 2, further enhancing its capabilities for multilingual understanding and data synthesis, catering specifically to research and educational purposes. Recent iterations, such as LLaMA3.2 (in April 2024), have introduced multimodal functionality, enabling the integration of textual and visual data for more comprehensive analyses [63]. In addition to its multimodal capabilities, newer versions like LLaMA3.1 and LLaMA3.3 have incorporated multilingual support, broadening their usability across diverse linguistic contexts [63]. This progression underscores the iterative refinement of generative AI models, focusing not only on enhancing performance but also on increasing accessibility and applicability in global and interdisciplinary research.

PaLM (Pathways Language Model), developed by Google in 2022, was designed to scale language understanding and generation across multiple domains. Leveraging Google’s Pathways system, PaLM enables large-scale model training while supporting multimodal capabilities [64,65]. In 2023, PaLM was incorporated into Google’s Bard chatbot and Gemini AI ecosystem, expanding its reach and practical use cases, especially for comprehensive tasks requiring both text and image processing. PaLM’s advancements in natural language understanding and its multimodal capabilities have positioned it as a model for educational and research-based applications [64].

### 1.2. Role of Multimodal AI in Ophthalmology

The role of multimodal AI in ophthalmology is particularly significant due to the field’s strong reliance on imaging data for diagnosis, monitoring, and treatment planning [66,67]. Ophthalmology extensively utilizes imaging modalities, such as fundus photography, optical coherence tomography (OCT), and fluorescein angiography (FFA), to detect and manage conditions like diabetic retinopathy, age-related macular degeneration, and glaucoma. Multimodal AI enhances imaging data analysis by integrating these visual inputs with other data sources, such as patient demographics, clinical histories, and biomarkers, to provide more accurate and comprehensive insights [68]. One of the key advantages of multimodal AI in imaging analysis is its ability to combine and interpret complex datasets simultaneously [29,69]. For example, while OCT provides high-resolution cross-sectional images of retinal layers, fundus photography offers a broader view of retinal structures. Multimodal AI can fuse these datasets to identify subtle patterns that may not be apparent when analyzed individually, thus improving diagnostic accuracy and sensitivity. Additionally, multimodal AI algorithms excel at handling the large volumes of imaging data typically generated in ophthalmology [70]. For instance, for detecting diabetic retinopathy, multimodal AI can cross-reference the imaging data with clinical information, such as HbA1c levels or systemic disease history, to provide more personalized assessments. Also, this integrative capability is particularly valuable for tracking disease progression [71]. Multimodal AI can analyze longitudinal imaging data to detect minute changes over time, allowing for earlier intervention and better prognostic predictions.

Multimodal AI can revolutionize imaging data analysis in ophthalmology by augmenting traditional diagnostic workflows, reducing human error, and providing deeper insights through the integration of diverse data types. Its application can not only enhances clinical decision-making, but also contribute to the ongoing development of precision medicine in ophthalmology.

### 1.3. Rapid Advancements in AI

The field of AI, particularly in the context of AI-based chatbots and related APIs, is characterized by rapid and continuous transformation [72,73]. This dynamic evolution is driven by the persistent efforts of developers, technology companies, and extensive research and development activities aimed at advancing the capabilities and efficiency of AI systems. Among the most noteworthy recent milestones are ChatGPT-4o, LLaMA3.2, Claude 3.5 Sonnet, and Grok-2, each of which has introduced various features that enhance the functionality, efficiency, and versatility of AI technologies. These developments highlight the increasing pace at which the field is moving, enabling more sophisticated tools, improved accuracy, and broader applicability across various domains. Grok-2, launched in August 2024 by xAI, builds on the foundation of its predecessor, Grok-1, by introducing enhanced conversational abilities and deeper integration with enterprise applications [60]. Notably, Grok-2 supports real-time interactions and is accessible on the X platform, making it particularly useful for professional and social ecosystems. In addition, Grok-2 includes multimodal capabilities, allowing it to process both text and visual inputs, further expanding its versatility and use cases in areas like content creation and marketing. Likewise, Claude 3.5 Sonnet, released on 20 June 2024 by Anthropic, represents a significant upgrade from Claude 3.0 [74]. While Claude 3.0 focused on ethical AI practices and natural language understanding, Claude 3.5 Sonnet introduces advanced visual problem-solving abilities alongside improved performance in reading comprehension, mathematics, and coding. Similarly, ChatGPT-4o, released in May 2024 by OpenAI, builds upon its predecessor, ChatGPT-4, by integrating advanced multimodal capabilities. This allows it to process both textual and visual inputs, enabling richer interactions and expanding its application across domains like healthcare and education. Moreover, ChatGPT-4o features enhanced reasoning capabilities, making it more effective at addressing complex queries and problem-solving tasks. Another significant development is the transition toward multimodal AI, exemplified by models such as LLaMA3.2, which integrates vision capabilities alongside text-based processing [63]. Multimodal AI, in particular, demonstrates an ability to combine textual and visual data inputs, which is of profound importance for domains heavily reliant on imaging, such as medical diagnostics [75]. These systems can analyze complex datasets, such as medical images paired with clinical notes, with improved accuracy and efficiency compared to single-modality approaches. The transition from earlier versions like LLaMA2 to more advanced iterations such as LLaMA3.2 underscores the iterative nature of these technologies, reflecting their ability to evolve and incorporate enhanced functionalities rapidly [75].

While this fast-paced evolution brings numerous advantages, including enhanced functionalities and expanded practical uses, it also presents significant challenges. One of the key issues is the potential obsolescence of academic studies, which could become outdated in a relatively short time due to the accelerated progression of AI technologies. As tools, models, and approaches in AI are updated or replaced at unprecedented rates, researchers face the challenge of ensuring that their work remains relevant and reflective of the current technological landscape. This underscores the importance of continuous monitoring, iterative revisions, and adaptability in academic research to keep pace with the advancements in this fast-changing domain. Consequently, scholars should adopt a forward-looking perspective and engage in ongoing updates to their findings and methodologies to sustain the impact and applicability of their contributions within the ever-evolving AI landscape.

## 2. Materials and Methods

“PubMed” and “Web of Science” medical databases were utilized for the literature search. The term “chatbot” was combined with keywords, such as “eye”, “eye disorders”, “eye disease”, “ocular”, “ophthalmology”, “ophthalmic”, and “ophthalmologist”. These keywords were selected based on relevant bibliographies and the latest literature. Additionally, manual searches of the initial results were performed to identify further relevant bibliographic references. Through this process, two additional references [67,76] were identified and included in this review, further enriching the comprehensiveness of the literature analyzed. No restrictions were placed on the earliest publication date, and the search was conducted up to November 2024.

Eligible studies were articles written in English. Exclusion criteria included non-English publications and studies with insufficient outcome data.

A total of 100 full-text articles were identified through the search. Of these, 44 articles were excluded after the initial screening due to duplication. The remaining 56 articles were assessed for eligibility through a full-text review. Following this evaluation, 43 articles (27 involving open-ended questions and 16 involving closed-ended questions) were deemed relevant and included in this narrative review. The selected publications were jointly reviewed and approved by all authors to ensure their relevance and alignment with this study’s objectives.

Open-ended questions were defined as those that allow users to provide free-text responses or engage in conversational exchanges without predefined constraints. In contrast, closed-ended questions were characterized by limited response options, such as multiple-choice. The justification for this classification lies in its ability to provide insight into the functional capabilities and limitations of chatbots in ophthalmology. Open-ended questions are critical for assessing a chatbot’s capacity for NLP and understanding nuanced patient responses. Meanwhile, closed-ended questions are essential for evaluating a chatbot’s accuracy. By clearly delineating these categories, this study provides a nuanced evaluation of chatbot performance, ensuring that their application aligns with the specific needs and challenges of ophthalmology.

## 3. Open-Ended Question Performance of Chatbots in Ophthalmology

The open-ended question studies in ophthalmology are detailed in Table 2.

### Opportunities and Challenges of Using Open-Ended Questions with Chatbots

Numerous studies have explored the usefulness of chatbots for diagnosing ophthalmologic diseases [37,40,51,70,77,78]. While chatbots often perform well with straightforward cases, they struggle with complex scenarios, frequently failing to provide comprehensive answers. Given the crucial role of imaging in ophthalmology, chatbots still face substantial challenges in image analysis; a recent study found they responded to image-based ophthalmologic cases with only 50.7% accuracy [70]. Studies on retinal disease, orbital and oculofacial disorders, glaucoma treatment, uveitis, and surgical planning have shown promising results [51,78,79,80,81,82]. Although no current studies focus on personalized treatment recommendations tailored to individual eye health profiles, an AI or chatbot with comprehensive access to EHRs could potentially provide more personalized diagnoses, recommendations, and treatment plans in the future. On the other hand, a study that examined ophthalmological advice generated by chatbots found that these tools can be useful and often provide results comparable to advice written by ophthalmologists [43]. Even more, recent AI tools (e.g., Bland AI, Retell AI, Vapi AI, and Air AI) are capable of making automated patient phone calls; however, they are not currently regarded as substitutes for professional ophthalmological consultations or examinations.

Patient education is a crucial component of treatment and a key responsibility of healthcare professionals. While the benefits of chatbots in this area are often debated, studies indicate they can be effective, beneficial, and safe [35,36,38,39,41,42,83,84,85]. Face-to-face patient–physician interactions remain essential, as these interactions often improve education and adherence by allowing healthcare providers to play an active role, receive real-time feedback, and better assess patient understanding. Still, chatbots might improve post-operative care and patient education. Particularly in busy healthcare systems, they could fill gaps by answering questions that arise at home that might otherwise go unaddressed [86]. Another example could be ophthalmic cancer patients, who may forget key information due to attentional limitations, a common phenomenon in cancer diagnoses. Here, chatbots trained in ophthalmic oncology could provide accessible, tailored support. This would not only improve understanding, but also empower patients to become active participants in their care [8].

**Table 2:** Chatbot studies conducted with open-ended questions in ophthalmology.

Study	Year	Objective(s)	Language	Method(s)	Chatbot Type(s)	Findings
Lyons et al. [87]	2024	To evaluate the triage performance of AI chatbots for ophthalmic conditions.	English	Primary outcomes were proportion of responses with the correct diagnosis listed in the top 3 possible diagnoses and proportion with correct triage urgency.	Bing Chat ChatGPT-4	The ophthalmologists in training, ChatGPT, and Bing Chat listed the appropriate diagnosis among the top 3 suggestions in 42 (95%), 41 (93%), and 34 (77%) cases, respectively. Triage urgency was appropriate in 38 (86%), 43 (98%), and 37 (84%) cases for ophthalmology trainees, ChatGPT, and Bing Chat, respectively.
Chen et al. [34]	2024	To assess the accuracy of assessment and plans generated by ChatGPT and evaluate ophthalmologists’ abilities to distinguish between responses.	English	Prompt engineering matched ChatGPT output to an ophthalmologist’s style. Masked discussions were evaluated by ophthalmologists, with confidence scored on a 5-point Likert scale.	ChatGPT-3.5	ChatGPT correctly identified the primary diagnosis in 15 of 17 (88.2%) cases. Ophthalmologists correctly identified the author in 77.9% ± 26.6% of the 13 included cases, with a mean Likert scale confidence rating of 3.6 ± 1.0.
Balas et al. [76]	2024	To explore the potential misuse of artificial intelligence (AI), specifically large language models (LLMs), in generating harmful content related to ocular warfare.	English	The study tested ChatGPT-4.0’s safeguards using adversarial prompts focused on hypothetical scenarios involving ocular damage by biological, chemical, and physical means.	ChatGPT-4	The AI gave detailed responses on using Onchocerca volvulus, methanol, mustard gas, and lasers for ocular harm. Despite safeguards, harmful information could still be accessed, revealing AI vulnerabilities.
Nikdel et al. [35]	2024	To assess the responses to frequently asked questions regarding two common pediatric ophthalmologic disorders, amblyopia and childhood myopia.	English	Twenty-seven questions about amblyopia and 28 questions about childhood myopia were asked of the ChatGPT twice (total of 110 questions).	ChatGPT-4	There was remarkable agreement (96.4%) between the two pediatric ophthalmologists on their assessment of the responses. The ChatGPT gave inappropriate responses to three questions about amblyopia (5.6%) and childhood myopia (5.4%).
Zandi et al. [20]	2024	To evaluate and compare responses to 40 critical ophthalmic triage cases questions.	English	Three ophthalmologists graded chatbot responses.	Bard ChatGPT-4	Chatbots were significantly better at ophthalmic triage than diagnosis, and GPT-4 performed better than Bard for appropriate triage recommendations, grader satisfaction for patient use, and lower potential harm rates.
Anguita et al. [8]	2024	To evaluate the accuracy of answers to common patient questions about choroidal melanoma.	English	Answers were reviewed by three ocular oncology experts and scored as accurate, partially accurate, or inaccurate.	ChatGPT-3.5 Bing DocsGPT	For medical advice questions, ChatGPT gave 92% accurate responses compared to 58% for Bing AI and DocsGPT. For pre-/post-op questions, 86%, 86%, and 73%, respectively.
Durmaz Engin et al. [36]	2024	To evaluate the effectiveness of responses to patient inquiries about retinopathy of prematurity.	English	The answers of LLMs for fifty real-life patient inquiries were assessed using a 5-point Likert scale by three ophthalmologists.	Bing Gemini ChatGPT-4	ChatGPT-4 outperformed BingAI and Gemini, scoring the highest with 5 points in 90% (45 out of 50) and achieving ratings of “agreed” or “strongly agreed” in 98% (49 out of 50) of responses.
Yalla et al. [83]	2024	The study evaluates glaucoma information from chatbots by comparing response accuracy, comprehensiveness, readability, and length with AAO materials.	English	Section headers from AAO glaucoma-related patient education brochures were adapted into question form and asked five times to each chatbot, and scored 1–5 by three glaucoma-trained ophthalmologists.	Bing Bard ChatGPT-4	Accuracy scores for AAO, ChatGPT, Bing, and Bard were 4.84, 4.26, 4.53, and 3.53, respectively. ChatGPT had the most comprehensive responses.
Huang et al. [71]	2024	To investigate the capability of ChatGPT for forecasting the conversion from OHT to glaucoma based on the OHTS.	English	They analyzed demographic, clinical, ocular, and VF data from OHTS participants to assess ChatGPT’s ability to predict conversion from OHT to glaucoma.	ChatGPT-3.5 ChatGPT-4	ChatGPT4.0 (and 3.5) demonstrated an accuracy of 75% (61%), AUC of 0.67 (0.62), sensitivity of 56% (64%), specificity of 78% (59%), and weighted F1 score of 0.77 (0.63) at predicting conversion to glaucoma 1 year before onset.
Shukla et al. [37]	2024	To compare the capabilities of chatbots at handling neuro-ophthalmological case scenarios.	English	Ten randomly chosen neuro-ophthalmological cases from a publicly accessible database were used to test accuracy and suitability.	Bing Gemini ChatGPT-3.5	All three chatbots achieved a 40% accuracy rate at diagnosis. For suitability, ChatGPT 3.5, Bing, and Gemini provided appropriate responses in 60%, 50%, and 50% of scenarios, respectively.
Cohen et al. [38]	2024	To identify the most common patient FAQs about cataracts and cataract surgery and evaluate the accuracy, safety, and readability of the answers.	English	Responses to the questions provided by Google and ChatGPT were evaluated by a panel of ophthalmologists for accuracy and safety.	ChatGPT-3.5	Responses to 20 patient FAQs generated by ChatGPT were significantly longer and written at a higher reading level than responses provided by Google, with an average grade level of 14.8 (college level).
Ahmed et al. [39]	2024	To assess the reliability of responses to inquiries posed by parents, focusing on pediatric ophthalmological and strabismus conditions.	English	All responses were scored on their response quality by two experienced pediatric ophthalmologists using a Likert-scale format.	ChatGPT-3.5	A total of 638 (78.09%) questions were scored to be perfectly correct, 156 (19.09%) were scored correct but incomplete, and only 23 (2.81%) were scored to be partially incorrect.
Schumacher et al. [80]	2024	A series of highly clinically relevant questions were asked to LLM regarding current uveitis cases.	English	The answers were classified on whether they were accurate and sufficient, partially accurate and sufficient, or inaccurate and insufficient.	ChatGPT 4.o	GPT 4.o was able to give accurate and sufficient answers in >80% of cases.
Rojas-Carabali et al. [40]	2024	To test the diagnostic accuracy of ChatGPT for various uveitis entities, comparing its performance to uveitis specialists.	English	They compared ChatGPT and five uveitis-trained ophthalmologists on 25 cases using new Uveitis Nomenclature guidelines, assessing diagnoses, differentials, and management.	ChatGPT-3.5 ChatGPT-4	Ophthalmologists excelled (60–92%) at likely diagnosis, exceeding AI (60%). Considering fully and partially accurate diagnoses, ophthalmologists achieved 76–100% success; AI attained 72%.
Carlà et al. [51]	2024	To define the capability of chatbots for analyzing retinal detachment cases and surgical planning.	English	Analysis of 54 retinal detachment records with the common opinion of three expert vitreoretinal surgeons.	Gemini ChatGPT-3.5 ChatGPT-4	ChatGPT-3.5, ChatGPT-4, and Google Gemini surgical choices agreed with those of vitreoretinal surgeons in 80%, 84%, and 70% of cases.
Tan et al. [79]	2024	To evaluate the accuracy, comprehensiveness, and safety of a publicly available LLM ChatGPT in the sub-domain of glaucoma.	English	They curated 24 clinically relevant glaucoma questions across diagnosis, treatment, surgeries, and ocular emergencies. Responses were graded by three glaucoma specialists.	ChatGPT-3.5	23 out of 24 (95.8%) of question–response pairs were rated as 3 or higher by all three experts.
Tailor et al. [41]	2024	To assess the quality, empathy, and safety of expert-edited LLM, human expert created, and LLM responses to common retina patient questions.	English	Responses to twenty-one common retina patient questions were generated by chatbots. Responses were graded for quality and empathy score, proportion of responses with incorrect information, likelihood to cause harm, and missing content by 13 retina specialists.	Bard Bing Claude 2 ChatGPT-3.5 ChatGPT-4	For quality, expert + AI (3.86 ± 0.85) performed the best overall, while GPT-3.5 (3.75 ± 0.79) was the top-performing LLM. ChatGPT-4 performed similarly to expert regarding inappropriate content, missing content, extent of possible harm, and likelihood of possible harm.
Maywood et al. [77]	2024	To determine how often ChatGPT is able to provide accurate and comprehensive information regarding clinical vitreoretinal scenarios.	English	Responses of forty open-ended clinical scenarios in vitreoretinal disease were graded on correctness and comprehensiveness by three blinded retina specialists.	ChatGPT-3.5	ChatGPT answered 83% of clinical scenarios (33/40) correctly but provided a comprehensive answer in only 52.5% of cases (21/40).
Mihalache et al. [68] *	2024	To assess the performance of chatbots at providing accurate diagnoses of retina teaching cases from OCTCases.	English	They prompted a custom chatbot with 69 retina cases containing multimodal ophthalmic images, asking it to provide the most likely diagnosis.	ChatGPT-4	For 69 retina cases collectively containing 139 ophthalmic images, the chatbot was able to provide a definitive, correct diagnosis for 35 (50.7%) cases.
Carlà et al. [78]	2024	To define the capability of chatbots at analyzing detailed glaucoma case descriptions and suggesting an accurate surgical plan.	English	Responses to 60 retrospective medical record cases by chatbots were used to assess the level of agreement with the consensus opinion of three glaucoma surgeons.	Gemini ChatGPT-4	ChatGPT’s surgical choice was consistent with that of glaucoma specialists in 35/60 cases (58%), compared to 19/60 (32%) for Gemini (*p* = 0.0001). Gemini was not able to complete the task in 16 cases (27%).
Yilmaz et al. [84]	2024	To assess whether chatbots enhance or disrupt education by offering interactive, accessible information and recommendations on cataract.	English	A descriptive comparative design was used to analyze quantitative data from frequently asked questions about cataracts answered by ChatGPT, Bard, Bing AI, and the AAO website.	Bing Bard ChatGPT-3.5	Chatbots scored higher than AAO website on cataract-related questions in terms of accuracy. For understandability and actionability, AAO website scored better than chatbots.
Milad et al. [88]	2024	To evaluate the proficiency of GPT-4 at answering questions about complex clinical ophthalmology cases.	English	Presented 422 *Journal of the American Medical Association* Ophthalmology Clinical Challenges, and prompted the model to determine the diagnoses.	ChatGPT-4	GPT-4 achieved mean accuracy of 48.0% (95% CI (43.1% to 52.9%)).
Lim et al. [42]	2023	To evaluate the performance of chatbots for delivering accurate responses to common myopia-related queries.	English	Thirty-one commonly asked myopia care-related questions were posed to the LLMs, and their responses were independently graded by three consultant-level pediatric ophthalmologists.	Bard ChatGPT-3.5 ChatGPT-4	ChatGPT-4 demonstrated superior accuracy, with 80.6% of responses rated as ‘good’, compared to 61.3% for ChatGPT-3.5 and 54.8% for Google Bard.
Bernstein et al. [43]	2023	To evaluate the quality of ophthalmology advice generated by an LLM chatbot in comparison with ophthalmologist-written advice.	English	Identification of chatbot and human answers on a 4-point scale and evaluation of responses.	ChatGPT-3.5	The mean (SD) accuracy for distinguishing between AI and human responses was 61.3% (9.7%). Of 800 evaluations of chatbot-written answers, 21.0% of answers were marked as human written, while 64.6% human-written answers were marked as AI written.
Potapenko et al. [89]	2023	Accuracy of patient information for common retinal diseases.	English	The accuracy of chatbot responses were rated on a Likert scale from 1 to 5 by 5 researchers.	ChatGPT-3.5	Of 100 responses, 45 were evaluated as very good. Of the remaining 46 responses, 26 were marked by minor non-harmful inaccuracies.
Rasmussen et al. [90]	2023	Accuracy of responses to typical patient-related questions on VKC.	English	Responses were rated on a Likert scale from 1 to 5 by two experts.	ChatGPT-3.5	Responses to treatment/prevention questions obtained lower scores than the rest. Some suggestions were inadequate, while some were considered harmful.
Momenaei et al. [85]	2023	To evaluate the appropriateness and readability of the medical knowledge provided by chatbots regarding common vitreoretinal surgeries.	English	Common questions about RD, MH, and ERM were created by authors, and two independent retina specialists graded the appropriateness of the responses.	ChatGPT-4	Responses were consistently appropriate for 84.6% (33/39), 92% (23/25), and 91.7% (22/24) of the questions related to RD, MH, and ERM, respectively.

* The study contained visual data. OHT: ocular hypertension; OHTS: ocular hypertension treatment study; AAO: American Academy of Ophthalmology; FAQs: frequently asked questions; LLM: large language model; PCCS: post-operative care chatbot system; RD: retinal detachment; MH: macular hole; ERM: epiretinal membrane.

Chatbots can significantly enhance triage efficiency and assist with differential diagnoses in ophthalmology [20,87]. These tools hold significant potential for assisting patients and triage staff; however, they are not a complete substitute for professional ophthalmic evaluation or advice [20,87].

Online-only access raises serious privacy concerns, emphasizing the need for robust encryption techniques [37,43,68,76]. Securing informed consent for any patient data shared with chatbots is crucial. Additionally, inputting protected health information into chatbots for medical advice risks severe and unpredictable consequences in the event of data breaches or hacking. However, chatbots that can be accessed locally and operate offline offer a potential solution to these privacy issues. By processing sensitive data entirely on secure local devices without transmitting them to external servers, offline-capable chatbots lower the risk of data breaches while maintaining the functionality required for clinical applications. Such models present an appealing alternative for healthcare environments where data security is paramount.

Chatbots offer valuable tools for translation and interpretation, helping to overcome language barriers and cultural differences in ophthalmology practices [91]. They also facilitate tasks like back-translation, enhancing academic work and broadening access to research in multiple languages. However, inconsistencies in chatbot responses across different languages present a significant concern [50]. It is well known that some academic publications are now written with the assistance of chatbots, raising ethical concerns. While chatbots are capable of generating responses that appear coherent and plausible, it is important to recognize that these outputs may contain factual inaccuracies and fabricated references—a phenomenon often referred to as “hallucination” [40,67,92]. Beyond these issues, this practice of drafting manuscripts using chatbots poses potential risks to the integrity of the academic community and the literature. While they can offer benefits when used responsibly, their potential harms should not be overlooked. Many academic journals now require authors to disclose any chatbot contributions at the end of an article. The International Committee of Medical Journal Editors (ICMJE) has included a section on the disclosure of the use of chatbots in academic writing in their latest recommendations, and state that the human authors are ultimately responsible for the contents [93].

Another concern regarding chatbots is whether they might violate the foundational principle of the AI and robotics industry: to avoid causing harm to humans or sharing information that could lead to harm [94]. Contrary to this expectation, chatbots have, in response to specific commands, provided information that could damage the eye. They have delivered detailed responses on using *Onchocerca volvulus* for biological harm, methanol and mustard gas for chemical injury, and lasers for physical damage—offering information that could be used for harmful ocular applications or warfare [76]. While most chatbots are programmed to avoid answering questions that could harm humans, reports indicate that certain prompts can bypass these safeguards. Despite these security measures, access to harmful information remains possible, highlighting the ongoing need for precautions.

## 4. Closed-Ended Question Performance of Chatbots in Ophthalmology

The close-ended question studies in ophthalmology are detailed in Table 3.

### Opportunities and Challenges of Using Close-Ended Questions with Chatbots

Questions that arise while studying a course or topic can be quickly addressed through chatbots, making a significant contribution to the learning and education process. Cost efficiency, 24/7 accessibility, and the capacity for personalized feedback further enhance chatbots’ educational advantages. In addition to answering questions, chatbots can explain why certain answers are correct and why others are incorrect, and they can also provide scientific references upon request. In this way, chatbots can enhance a user’s knowledge of specific subjects and make it easier to access accurate information. But, chatbots can occasionally hinder the acquisition of scientific results by generating hallucinations and fabricating references [67,92]. Furthermore, image analysis remains a challenge for chatbots when responding to questions that include images, indicating a need for further advancement [67,70]. However, the potential for improvement in this area is promising.

Conversely, the rapid response time of the chatbots raises concern about their misuse during exams, particularly in MCQ assessments. Unauthorized access to chatbots during exams could compromise academic integrity, leading to serious irregularities, grievances, and potential injustices. Addressing this issue requires robust security measures, such as monitoring technological devices and implementing stricter exam protocols. These measures must also be accompanied by a strong emphasis on ethical accountability. Beyond security concerns, chatbots also face limitations in educational contexts. These include inconsistent performance across different versions, over-reliance on their capabilities, limited ability to interpret nuances in complex MCQs, practical application constraints, and privacy concerns. Furthermore, while emotional intelligence is a vital component of effective education, chatbots currently lack the depth of emotional understanding needed to foster the human connection essential for meaningful learning experiences [102,103,104].

On the other hand, some chatbots are equipped with security measures that prevent them from answering MCQs that involve offering medical treatment suggestions and recommendations. In such cases, responses like “I’m only a language model”, “I’m a text-based AI, and that is outside of my capabilities”, or “As a language model, I’m not able to assist with that”, are typically provided [67,90].

Another challenge is that most chatbots require a monthly or annual paid subscription. While nearly all offer free versions, these often provide access only to older versions and impose significant time and word limits, restricting the scope of information accessible in the free tier. Another disadvantage is that chatbot responses may vary based on the training data and version of the chatbot, potentially leading to inconsistencies in the answers over time.

## 5. Discussion and Expert Opinion

AI-based LLM chatbots have the potential to become valuable tools for educational, research, and clinical applications in ophthalmology. The current applications and advantages of AI-based LLM chatbots encompass disease diagnosis support, symptom assessment and triage, patient education and engagement, preparation assistance for ophthalmic board exams, surgical and treatment guidance, literature analysis and review, and drafting responses to patient inquiries.

The image evaluation capabilities of chatbots are currently only partially effective, yet they hold promising potential [67,70]. Given that most ophthalmological examination techniques rely heavily on imaging and its interpretation, advancing these capabilities is critical. Enhancing the diagnostic accuracy of LLMs could be achieved by integrating image-based deep learning algorithms, allowing for the simultaneous contextual interpretation of both textual and visual inputs. This integration could significantly boost diagnostic precision and provide deeper clinical insights.

Currently, clinicians are raising concerns about the reliability, limited knowledge or training of chatbots, implementation costs, ethical concerns, and the absence of regulatory approval for integrating AI-based LLM chatbots into routine practice [105,106,107,108,109]. Tackling these challenges requires essential collaboration between chatbot developers and healthcare providers, ongoing professional training, strict regulatory oversight, and the establishment and enforcement of relevant ethical guidelines and regulations. Clinical studies are advancing rapidly to address these barriers, with the expectation that AI will become increasingly integrated into clinical practice. Once these barriers are overcome, AI-based tools will hold promise for enhancing early diagnosis, enabling personalized treatment planning, assisting with surgical procedures, supporting patient follow-up and monitoring, and minimizing medical errors linked to human factors. However, additional challenges may arise, such as potential harm to the patient–physician relationship and trust, loss of the human touch in patient care, liability issues from over reliance on technology, and risks of misinterpretation [110,111,112]. It remains an open question whether accountability for AI misinterpretations or AI-induced malpractice should rest with the physician, the AI system, its developers, or a shared responsibility. This calls for dialogue between physicians, AI developers, and national and international legislators, as well as with regulatory, judicial, and insurance systems.

Translation and linguistic reviews of academic research can indeed benefit from the use of chatbots. However, beyond the challenges noted, there is the potential for misuse in academic publishing, with manuscripts generated entirely by chatbots being submitted to journals for publication. Similarly, graduate theses may also be produced via chatbots, posing risks to academic rigor and originality. While chatbots offers significant benefits, it is essential to use them judiciously, acknowledging their limitations and ensuring that their outputs are critically evaluated to maintain academic integrity [46,47]. The development of binding principles for the scientific community seems appropriate and necessary to ensure continued trust in scientific publications.

The potential benefits of the current trends and capabilities of LLMs in ophthalmology include remote monitoring and teleconsultation, accessible support for clinicians in busy clinics, the provision of accurate and evidence-based information, ongoing education, prognosis estimation, and contact lens and refractive correction suggestions. They could also have valuable roles as research or administrative assistants; aid in clinical documentation and report generation, integration with EHRs, clinical decision-making and differential diagnosis support, image analysis, interpretation and recommendations, pre-operative patient counseling; and function as enhanced tools for both surgical and medical education [66]. These developments outline a promising future for LLMs in ophthalmology. In conclusion, AI-based LLM chatbots hold transformative potential for ophthalmology, but addressing their current limitations and ethical concerns is crucial for their safe and effective integration into clinical practice.

Last but not least, the rapid evolution of AI technologies offers numerous advantages but also poses challenges, particularly the risk of academic studies becoming outdated quickly. With tools and models evolving at unprecedented rates, researchers must continuously adapt their work to remain relevant. This highlights the need for ongoing monitoring, iterative revisions, and a forward-looking approach to ensure the impact and applicability of scholarly contributions in this fast-changing field.

### 5.1. The Future of AI-Based LLMs

As AI continues its rapid evolution, its influence on the future of various professions, including medicine, is becoming increasingly apparent. LLMs are transformative tools in ophthalmology, improving patient communication, diagnosis, and education [113]. To ensure these tools are integrated responsibly, the development of standardized frameworks and guidelines will be critical, providing a roadmap for maintaining ethical and legal compliance. Looking ahead, we can envision AI-based interpretations and recommendations becoming seamlessly integrated into clinical workflows. For example, results from imaging modalities like OCT and OCTA could be transmitted directly to physicians, augmenting their diagnostic capabilities. Also, when multimodality instruments are interconnected, they can acquire, collate, and interpret data from fundus photography, FFA, perimetry, autorefractometry, corneal topography, pachymetry, tonometry, OCT, and OCTA. Similarly, EHRs could evolve to generate personalized ophthalmological recommendations by analyzing patient-specific data. Going even further, patients may soon be able to transmit their symptoms and hospital data to AI-based diagnostic chatbots from their homes, hospital entry points, or electronic diagnostic centers, receiving both a diagnosis and treatment recommendations in return. While these advancements may seem ambitious, they are no longer confined to the realm of science fiction and could become reality in the near future.

### 5.2. Limitations

The narrative and non-systematic nature of this review, together with the fact that it was conducted using only two scientific databases (PubMed and Web of Science), could be considered limitations of this study. Additionally, despite a thorough search and screening process, some relevant chatbot studies might have been inadvertently overlooked. Furthermore, as the majority of the included studies focus on widely adopted and mainstream chatbots, this review may provide limited insights into less prominent or emerging chatbots and API technologies. Also, the narrative nature of this review inherently affects the robustness and generalizability of the findings, as it lacks the structured methodology and statistical rigor of systematic reviews or meta-analyses. Lastly, the absence of a formal bias analysis, which is not commonly included in narrative reviews, may also be considered a limitation of this study. While this review provides a broad overview of chatbots with open- and close-ended questions applications in ophthalmology, its limitations highlight the need for future studies to adopt more rigorous methodologies, include a wider range of databases, and focus on emerging chatbot technologies to ensure a more comprehensive and balanced understanding of the field.

### 5.3. Future Directions

In light of the scope and limitations of this narrative review, several gaps in the current research and opportunities for further investigation emerge. One of the most significant areas requiring exploration is the potential role of AI in fostering digital therapeutic alliances. A digital therapeutic alliance refers to the relationship between a patient and a digital tool, such as a chatbot, which aims to build trust, enhance patient engagement, and improve adherence to medical advice. Understanding how chatbots and AI-based systems can emulate or support the relational aspects of traditional patient–provider interactions is crucial for maximizing their impact in ophthalmology.

Another promising avenue for future research lies in the development and application of multimodal AI in ophthalmology. Multimodal AI systems, capable of integrating and analyzing diverse data types, such as imaging, clinical history, and genetic information, hold significant potential for advancing precision medicine in this field. Investigating how these systems could enhance diagnostic accuracy, predict disease progression, and personalize treatments will be vital. Furthermore, understanding the limitations, ethical considerations, and practical challenges of implementing multimodal AI in clinical practice should be a key focus.

Additionally, there is a need to evaluate the long-term effectiveness and reliability of AI-driven chatbots in ophthalmology. Future studies should assess their ability to provide accurate information, support decision-making, and improve clinical outcomes over extended periods and across diverse patient populations. Exploring their integration with EHRs and their impact on healthcare workflows could further inform their scalability and real-world applications.

Lastly, as AI technology evolves, interdisciplinary research combining expertise from ophthalmology, computer science, ethics, and behavioral science will be essential. Such collaborations could address gaps in the usability, accessibility, and inclusivity of AI systems, ensuring they meet the needs of diverse populations and healthcare settings. By addressing these gaps, future research could unlock the full potential of AI in ophthalmology, leading to more effective, equitable, and innovative solutions for patient care.

## 6. Conclusions

Chatbots continue to evolve at an impressive pace, driven primarily by human innovation and iterative updates. While their capabilities have expanded significantly, particularly in answering both open-ended and closed-ended questions, they come with a balance of opportunities and challenges. To harness their potential effectively, collaboration between chatbot developers and healthcare providers is essential, ensuring that these tools are aligned with the needs of medical practice as well as with patient preferences and legislative frameworks. Despite their advancements, current chatbot models still lack the depth of knowledge and contextual awareness needed to fully replicate the expertise of a human physician, particularly in complex medical fields like ophthalmology. As their development continues, addressing these limitations will be crucial to integrating chatbots responsibly into clinical workflows, maintaining both safety and ethical standards. The future of chatbots in healthcare holds great promise, but their role must remain complementary to, rather than a replacement for, human expertise.

## Figures and Tables

**Table 1:** Available chatbots and related application programming interface tools and their features.

Model	Affiliation/Company	Release Date	Access	Multimodal Capability		Description
				Text	Visual	
Aeyeconsult	Aeye Health	2020	https://www.aeyeconsult.com/	✓	✓	AI tool for automated eye disease detection. Powered by GPT-4o and LangChainAI.
Bard	Google	March 2023	https://bard.google.com/ Free with Google account but currently unavailable after Gemini.	✓	Partial	AI chatbot integrated into Google Search, answering questions conversationally.
Bing	Microsoft	February 2023	https://www.bing.com/chat Free via Microsoft Edge and Bing application but currently unavailable after Copilot.	✓	✓	AI-powered search assistant integrated into Bing, providing conversational search results.
ChatGPT-3.5	OpenAI	November 2022	https://chat.openai.com/ Currently unavailable after ChatGPT-4.	✓	X	Language model offering conversational AI capabilities with general knowledge. The GPT3.5 legacy (free version) is much slower than GPT3.5 Default (GPT Plus).
ChatGPT-4	OpenAI	March 2023	https://chat.openai.com/ Available via ChatGPT Plus.	✓	✓	Advanced language model with improved accuracy and reasoning over GPT-3.5.
ChatGPT-4 omni	OpenAI	May 2024	https://chat.openai.com/ Available via ChatGPT Plus.	✓	✓	Multimodal version of GPT-4.
Claude	Anthropic	March 2023	https://claude.ai/ Limited access via partnerships and API.	✓	X	AI assistant focused on safety and helpfulness, named after Claude Shannon. Claude 3.5 Sonnet and Claude 3 Opus are available.
Cohere	Cohere	November 2022	https://cohere.com API access with subscription.	✓	X	Language AI platform offering NLP models for text generation and understanding.
Copilot	Microsoft	March 2023	https://copilot.microsoft.com Integrated into Microsoft 365 apps.	✓	X	AI assistant for productivity, aiding in tasks like writing, coding, and data analysis.
Falcon	Technology Innovation Institute	May 2023	https://falconllm.tii.ae/ Open source, available for commercial use.	✓	X	High-performance language model designed for various NLP tasks.
Gemini	Google DeepMind	December 2023	https://gemini.google.com Limited access via select partnerships with Google account.	✓	✓	Advanced AI combining language models and reinforcement learning for enhanced reasoning capabilities.
Grok	xAI	November 2023	https://x.ai/ Accessible on the X platform (formerly Twitter).	✓	✓	Grok-2 supports real-time interactions on the X platform. Grok-2 was released in August 2024.
LLaMA	Meta AI	February 2023	https://www.llama.com/ Research access upon request.	✓	X	Open-access language model designed for research in natural language understanding. LLaMA3.2 with multimodal and Llama 3.3 and 3.1 with multilingual support.
OpenAI o1	OpenAI	September 2024	https://chat.openai.com/ Available via ChatGPT Plus.	✓	X	o1 spends time “thinking” before it answers, making it more effective at complex reasoning tasks, science, and programming.
PaLM	Google	April 2022	https://ai.google/discover/palm2/ Limited access via API and partnerships.	✓	X	Large-scale language model for diverse natural language processing tasks.
Perplexity	Perplexity AI	August 2022	https://www.perplexity.ai/ Free and subscription-based plans.	✓	X	AI-powered search engine providing conversational answers with cited sources.

Chatbots are listed in alphabetical order.

**Table 3:** Chatbot studies conducted with close-ended questions in ophthalmology.

Study	Year	Objective(s)	Test Language	Method(s)	Data		Chatbot Type(s)	Accuracy	Other Findings
					Text	Visual			
Mihalache et al. [70]	2024	Response accuracy for image- and nonimage-based questions	English	429 multiple-choice questions from 136 ophthalmic cases and 448 images from OCTCase	✓	✓	ChatGPT-4	70%	The chatbot achieved a better performance on nonimage-based questions compared with image-based questions (82% vs. 65%).
Antaki et al. [95]	2024	Accuracy of LLM models with varying temperatures	English	260-question multiple-choice question sets from BCSC Self-Assessment Program and the OphthoQuestions question banks	✓	X	ChatGPT-3.5 GPT-4-0 GPT-4-0.3 GPT-4-0.7 GPT-4-1	58.8% and 50.4% 76.2% and 67.3% 75.8% and 70.0% 75.8% and 68.5% 76.5% and 66.9%	GPT-4-0.3’s performance was superior to humans’ on the BCSC (75.8% vs. 73.3%) and OphthoQuestions (70.0% vs. 63.0%), but the difference was not statistically significant.
Sabaner et al. [67]	2024	Response accuracy for three consecutive attempts	Swedish English	134 ophthalmology-related multiple-choice questions from Swedish proficiency test for medicine	✓	✓	ChatGPT-4o Gemini 3.5	94% and 95.5% 88.1% and 88.1%	In the final attempt, compared to the first and second attempts, a generally increasing, but not statistically significant, accuracy rate was observed.
Singer et al. [56]	2024	Response accuracy	English	260 questions from OphthoQuestions.com	✓	X	Aeyeconsult ChatGPT-4	83.4% 69.2%	Aeyeconsult also had fewer instances of no answer and multiple answers.
Fowler et al. [96]	2024	Response accuracy	English	49 questions from 2020 FRCOphth part 1 Exam and 385 questions from MCQs for FRCOpth part 1	✓	X	Bard ChatGPT-4	44.9% and N/A 85.7% and 69.9%	ChatGPT demonstrated a strong performance, surpassing historical human pass marks and examination performance, while Bard underperformed.
Mihalache et al. [50]	2024	Response accuracy in different countries	English	150 text-based multiple-choice questions from EyeQuiz	✓	X	Bard Gemini	71% 71%	Subtle variability was noted in the performance of the chatbots across different countries (US, Vietnam, Brazil, and the Netherlands).
Tao et al. [97]	2024	Response accuracy, readability score, and the resources referenced	English	913 questions from the Academy’s Basic and Clinical Science Collection	✓	X	Bing Chat ChatGPT-3.5	73.60% 59.69%	Bing Chat showed significantly better readability than ChatGPT-3.5, averaging a reading level of grade 11.4 versus 12.4. Bing Chat provided at least one citation to 836 of the 913 questions.
Botross et al. [29]	2024	Response accuracy	English	250 multiple-choice questions from the “BoardVitals” ophthalmology exam question bank	✓	✓	Bard	62.4%	While the majority of questions were entered with minimal difficulty, not all questions could be processed by Bard.
Haddad et al. [98]	2024	Response accuracy	English	132 questions from USMLE steps 1, 2, 3; and 248 questions from Ophthalmology Board Review Q&A	✓	X	ChatGPT-3.5 ChatGPT-4	55% 70%	The coefficient of correlation (r) between ChatGPT answering correctly and human users answering correctly was 0.21 (*p* = 0.01) for GPT-3.5, as compared to −0.31 (*p* < 0.001) for GPT-4.0.
Cai et al. [99]	2023	Response accuracy, performance on subcategories, and hallucination frequency	English	250 questions from the BSCS Self-Assessment Program	✓	✓	Bing Chat ChatGPT-3.5 ChatGPT-4	71.2% 58.8% 71.6%	ChatGPT-3.5 had the highest rate of hallucinations and nonlogical reasoning (42.4%), followed by ChatGPT-4.0 (18.0%) and Bing Chat (25.6%).
Panthier et al. [32]	2023	Response accuracy and time taken to answer each question	French	French version of the EBO examination (6785 questions)	✓	X	ChatGPT-4	91.2%	The AI model was also able to answer the questions rapidly, taking only a fraction of the time needed by human being (on the order of one second).
Antaki et al. [100]	2023	Response accuracy	English	260 questions from the BCSC and OphthoQuestions	✓	X	ChatGPT Legacy ChatGPT Plus	55.8% and 42.7% 59.4% and 49.2%	Legacy model showed that the examination section followed by question difficulty were most predictive of ChatGPT’s answer accuracy.
Taloni et al. [92]	2023	Response accuracy and comparison with humans	English	1023 questions from BCSC Self-Assessment Program	✓	X	ChatGPT-3.5 ChatGPT-4	65.9% 82.4%	Humans achieved 75.7% accuracy. ChatGPT is still limited by inconsistency across different practice areas, especially when it comes to surgery.
Moshirfar et al. [69]	2023	Response accuracy and comparison with humans	English	467 ophthalmology questions from the StatPearls question bank	✓	✓	ChatGPT-3.5 ChatGPT-4	55.5% 73.2%	Humans achieved 58.3% accuracy. GPT-4 consistently performed better than both GPT-3.5 and humans on level-two, -three, and -four questions.
Sakai et al. [33]	2023	Response accuracy and comparison with humans	Japanese	500 questions from board examinations for specialists in the Japanese Ophthalmology Society	✓	X	ChatGPT-3.5 ChatGPT-4	22.4% 45.8%	Humans achieved 65.7% accuracy. ChatGPT-4 correctly answered 46.2% of questions with few-shot prompting.
Mihalache et al. [101]	2023	Response accuracy on different dates	English	125 questions from OphthoQuestions.com	✓	X	ChatGPT-3.5	46%	After one month, ChatGPT provided a correct response to 73 of 125 multiple-choice questions (58%).

BCSC: Basic and clinical science course; LLM: large language model, EBO: European Board of Ophthalmology, USMLE: United States Medical Licensing Examination; FRCOphth: Fellowship of the Royal College of Ophthalmologists.

## Data Availability

Not applicable.

## References

[1] Wartman S.A., Donald Combs C. (2018). Medical Education Must Move from the Information Age to the Age of Artificial Intelligence. Acad. Med..

[2] Buabbas A.J., Miskin B., Alnaqi A.A., Ayed A.K., Shehab A.A., Syed-Abdul S., Uddin M. (2023). Investigating Students’ Perceptions towards Artificial Intelligence in Medical Education. Healthcare.

[3] Ting D.S.J., Tan T.F., Ting D.S.W. (2024). ChatGPT in Ophthalmology: The Dawn of a New Era?. Eye.

[4] Halaweh M. (2023). ChatGPT in Education: Strategies for Responsible Implementation. Contemp. Educ. Technol..

[5] Gill S.S., Xu M., Patros P., Wu H., Kaur R., Kaur K., Fuller S., Singh M., Arora P., Parlikad A.K. (2024). Transformative Effects of ChatGPT on Modern Education: Emerging Era of AI Chatbots. Internet Things Cyber-Phys. Syst..

[6] Labadze L., Grigolia M., Machaidze L. (2023). Role of AI Chatbots in Education: Systematic Literature Review. Int. J. Educ. Technol. High. Educ..

[7] Ferro Desideri L., Roth J., Zinkernagel M., Anguita R. (2023). Application and Accuracy of Artificial Intelligence-Derived Large Language Models in Patients with Age Related Macular Degeneration. Int. J. Retin. Vitr..

[8] Anguita R., Downie C., Ferro Desideri L., Sagoo M.S. (2024). Assessing Large Language Models’ Accuracy in Providing Patient Support for Choroidal Melanoma. Eye.

[9] Hirani R., Noruzi K., Khuram H., Hussaini A.S., Aifuwa E.I., Ely K.E., Lewis J.M., Gabr A.E., Smiley A., Tiwari R.K. (2024). Artificial Intelligence and Healthcare: A Journey through History, Present Innovations, and Future Possibilities. Life.

[10] Tlili A., Shehata B., Adarkwah M.A., Bozkurt A., Hickey D.T., Huang R., Agyemang B. (2023). What If the Devil Is My Guardian Angel: ChatGPT as a Case Study of Using Chatbots in Education. Smart Learn. Environ..

[11] Biswas S., Davies L.N., Sheppard A.L., Logan N.S., Wolffsohn J.S. (2024). Utility of Artificial Intelligence-based Large Language Models in Ophthalmic Care. Ophthalmic Physiol. Opt..

[12] Zou M., Chen A., Liu Z., Jin L., Zheng D., Congdon N., Jin G. (2024). The Burden, Causes, and Determinants of Blindness and Vision Impairment in Asia: An Analysis of the Global Burden of Disease Study. J. Glob. Health.

[13] Steinmetz J.D., Bourne R.R.A., Briant P.S., Flaxman S.R., Taylor H.R.B., Jonas J.B., Abdoli A.A., Abrha W.A., Abualhasan A., Abu-Gharbieh E.G. (2021). Causes of Blindness and Vision Impairment in 2020 and Trends over 30 Years, and Prevalence of Avoidable Blindness in Relation to VISION 2020: The Right to Sight: An Analysis for the Global Burden of Disease Study. Lancet Glob. Health.

[14] Varma R., Vajaranant T.S., Burkemper B., Wu S., Torres M., Hsu C., Choudhury F., McKean-Cowdin R. (2016). Visual Impairment and Blindness in Adults in the United States. JAMA Ophthalmol..

[15] Bourne R., Steinmetz J.D., Flaxman S., Briant P.S., Taylor H.R., Resnikoff S., Casson R.J., Abdoli A., Abu-Gharbieh E., Afshin A. (2021). Trends in Prevalence of Blindness and Distance and near Vision Impairment over 30 Years: An Analysis for the Global Burden of Disease Study. Lancet Glob. Health.

[16] Tham Y.-C., Li X., Wong T.Y., Quigley H.A., Aung T., Cheng C.-Y. (2014). Global Prevalence of Glaucoma and Projections of Glaucoma Burden through 2040. Ophthalmology.

[17] Wong W.L., Su X., Li X., Cheung C.M.G., Klein R., Cheng C.-Y., Wong T.Y. (2014). Global Prevalence of Age-Related Macular Degeneration and Disease Burden Projection for 2020 and 2040: A Systematic Review and Meta-Analysis. Lancet Glob. Health.

[18] Resnikoff S., Lansingh V.C., Washburn L., Felch W., Gauthier T.-M., Taylor H.R., Eckert K., Parke D., Wiedemann P. (2020). Estimated Number of Ophthalmologists Worldwide (International Council of Ophthalmology Update): Will We Meet the Needs?. Br. J. Ophthalmol..

[19] Bhai Shah A.N., Patel N., Dave J.A., Aluvalu R. (2023). Role of Artificial Intelligence and Neural Network in the Health-Care Sector. Computational Intelligence in Medical Decision Making and Diagnosis.

[20] Zandi R., Fahey J.D., Drakopoulos M., Bryan J.M., Dong S., Bryar P.J., Bidwell A.E., Bowen R.C., Lavine J.A., Mirza R.G. (2024). Exploring Diagnostic Precision and Triage Proficiency: A Comparative Study of GPT-4 and Bard in Addressing Common Ophthalmic Complaints. Bioengineering.

[21] Kung T.H., Cheatham M., Medenilla A., Sillos C., De Leon L., Elepaño C., Madriaga M., Aggabao R., Diaz-Candido G., Maningo J. (2023). Performance of ChatGPT on USMLE: Potential for AI-Assisted Medical Education Using Large Language Models. PLoS Digit. Health.

[22] Sadeq M.A., Ghorab R.M.F., Ashry M.H., Abozaid A.M., Banihani H.A., Salem M., Aisheh M.T.A., Abuzahra S., Mourid M.R., Assker M.M. (2024). AI Chatbots Show Promise but Limitations on UK Medical Exam Questions: A Comparative Performance Study. Sci. Rep..

[23] Cheung B.H.H., Lau G.K.K., Wong G.T.C., Lee E.Y.P., Kulkarni D., Seow C.S., Wong R., Co M.T.H. (2023). ChatGPT versus Human in Generating Medical Graduate Exam Multiple Choice Questions—A Multinational Prospective Study (Hong Kong S. A.R., Singapore, Ireland, and the United Kingdom). PLoS ONE.

[24] Liu M., Okuhara T., Chang X., Shirabe R., Nishiie Y., Okada H., Kiuchi T. (2024). Performance of ChatGPT Across Different Versions in Medical Licensing Examinations Worldwide: Systematic Review and Meta-Analysis. J. Med. Internet Res..

[25] Huwiler J., Oechslin L., Biaggi P., Tanner F.C., Wyss C.A. (2024). Experimental Assessment of the Performance of Artificial Intelligence in Solving Multiple-Choice Board Exams in Cardiology. Swiss Med. Wkly..

[26] Kim S.E., Lee J.H., Choi B.S., Han H.-S., Lee M.C., Ro D.H. (2024). Performance of ChatGPT on Solving Orthopedic Board-Style Questions: A Comparative Analysis of ChatGPT 3.5 and ChatGPT 4. Clin. Orthop. Surg..

[27] Samman L., Akuffo-Addo E., Rao B. (2024). The Performance of Artificial Intelligence Chatbot (GPT-4) on Image-Based Dermatology Certification Board Exam Questions. J. Cutan. Med. Surg..

[28] Oztermeli A.D., Oztermeli A. (2023). ChatGPT Performance in the Medical Specialty Exam: An Observational Study. Medicine.

[29] Botross M., Mohammadi S.O., Montgomery K., Crawford C. (2024). Performance of Google’s Artificial Intelligence Chatbot “Bard” (Now “Gemini”) on Ophthalmology Board Exam Practice Questions. Cureus.

[30] Thibaut G., Dabbagh A., Liverneaux P. (2024). Does Google’s Bard Chatbot Perform Better than ChatGPT on the European Hand Surgery Exam?. Int. Orthop..

[31] Long C., Lowe K., Zhang J., dos Santos A., Alanazi A., O’Brien D., Wright E.D., Cote D. (2024). A Novel Evaluation Model for Assessing ChatGPT on Otolaryngology–Head and Neck Surgery Certification Examinations: Performance Study. JMIR Med. Educ..

[32] Panthier C., Gatinel D. (2023). Success of ChatGPT, an AI Language Model, in Taking the French Language Version of the European Board of Ophthalmology Examination: A Novel Approach to Medical Knowledge Assessment. J. Fr. Ophtalmol..

[33] Sakai D., Maeda T., Ozaki A., Kanda G.N., Kurimoto Y., Takahashi M. (2023). Performance of ChatGPT in Board Examinations for Specialists in the Japanese Ophthalmology Society. Cureus.

[34] Chen J.S., Reddy A.J., Al-Sharif E., Shoji M.K., Kalaw F.G.P., Eslani M., Lang P.Z., Arya M., Koretz Z.A., Bolo K.A. (2024). Analysis of ChatGPT Responses to Ophthalmic Cases: Can ChatGPT Think like an Ophthalmologist?. Ophthalmol. Sci..

[35] Nikdel M., Ghadimi H., Tavakoli M., Suh D.W. (2024). Assessment of the Responses of the Artificial Intelligence–Based Chatbot ChatGPT-4 to Frequently Asked Questions About Amblyopia and Childhood Myopia. J. Pediatr. Ophthalmol. Strabismus.

[36] Durmaz Engin C., Karatas E., Ozturk T. (2024). Exploring the Role of ChatGPT-4, BingAI, and Gemini as Virtual Consultants to Educate Families about Retinopathy of Prematurity. Children.

[37] Shukla R., Mishra A.K., Banerjee N., Verma A. (2024). The Comparison of ChatGPT 3.5, Microsoft Bing, and Google Gemini for Diagnosing Cases of Neuro-Ophthalmology. Cureus.

[38] Cohen S.A., Brant A., Fisher A.C., Pershing S., Do D., Pan C. (2024). Dr. Google vs. Dr. ChatGPT: Exploring the Use of Artificial Intelligence in Ophthalmology by Comparing the Accuracy, Safety, and Readability of Responses to Frequently Asked Patient Questions Regarding Cataracts and Cataract Surgery. Semin. Ophthalmol..

[39] Ahmed H.S., Thrishulamurthy C.J. (2024). Evaluating ChatGPT’s Efficacy and Readability to Common Pediatric Ophthalmology and Strabismus-Related Questions. Eur. J. Ophthalmol..

[40] Rojas-Carabali W., Cifuentes-González C., Wei X., Putera I., Sen A., Thng Z.X., Agrawal R., Elze T., Sobrin L., Kempen J.H. (2023). Evaluating the Diagnostic Accuracy and Management Recommendations of ChatGPT in Uveitis. Ocul. Immunol. Inflamm..

[41] Tailor P.D., Dalvin L.A., Chen J.J., Iezzi R., Olsen T.W., Scruggs B.A., Barkmeier A.J., Bakri S.J., Ryan E.H., Tang P.H. (2024). A Comparative Study of Responses to Retina Questions from Either Experts, Expert-Edited Large Language Models, or Expert-Edited Large Language Models Alone. Ophthalmol. Sci..

[42] Lim Z.W., Pushpanathan K., Yew S.M.E., Lai Y., Sun C.H., Lam J.S.H., Chen D.Z., Goh J.H.L., Tan M.C.J., Sheng B. (2023). Benchmarking Large Language Models’ Performances for Myopia Care: A Comparative Analysis of ChatGPT-3.5, ChatGPT-4.0, and Google Bard. eBioMedicine.

[43] Bernstein I.A., Zhang Y.V., Govil D., Majid I., Chang R.T., Sun Y., Shue A., Chou J.C., Schehlein E., Christopher K.L. (2023). Comparison of Ophthalmologist and Large Language Model Chatbot Responses to Online Patient Eye Care Questions. JAMA Netw. Open.

[44] Lo C.K. (2023). What Is the Impact of ChatGPT on Education? A Rapid Review of the Literature. Educ. Sci..

[45] Shemer A., Cohen M., Altarescu A., Atar-Vardi M., Hecht I., Dubinsky-Pertzov B., Shoshany N., Zmujack S., Or L., Einan-Lifshitz A. (2024). Diagnostic Capabilities of ChatGPT in Ophthalmology. Graefe’s Arch. Clin. Exp. Ophthalmol..

[46] Albadarin Y., Saqr M., Pope N., Tukiainen M. (2024). A systematic literature review of empirical research on ChatGPT in education. Discov. Educ..

[47] Tiwari C.K., Bhat M.A., Khan S.T., Subramaniam R., Khan M.A.I. (2024). What Drives Students toward ChatGPT? An Investigation of the Factors Influencing Adoption and Usage of ChatGPT. Interact. Technol. Smart Educ..

[48] Yu H. (2024). The Application and Challenges of ChatGPT in Educational Transformation: New Demands for Teachers’ Roles. Heliyon.

[49] Masalkhi M., Ong J., Waisberg E., Lee A.G. (2024). Google DeepMind’s Gemini AI versus ChatGPT: A Comparative Analysis in Ophthalmology. Eye.

[50] Mihalache A., Grad J., Patil N.S., Huang R.S., Popovic M.M., Mallipatna A., Kertes P.J., Muni R.H. (2024). Google Gemini and Bard Artificial Intelligence Chatbot Performance in Ophthalmology Knowledge Assessment. Eye.

[51] Carlà M.M., Gambini G., Baldascino A., Giannuzzi F., Boselli F., Crincoli E., D’Onofrio N.C., Rizzo S. (2024). Exploring AI-Chatbots’ Capability to Suggest Surgical Planning in Ophthalmology: ChatGPT versus Google Gemini Analysis of Retinal Detachment Cases. Br. J. Ophthalmol..

[52] Roos J., Kasapovic A., Jansen T., Kaczmarczyk R. (2023). Artificial Intelligence in Medical Education: Comparative Analysis of ChatGPT, Bing, and Medical Students in Germany. JMIR Med. Educ..

[53] Morreel S., Verhoeven V., Mathysen D. (2024). Microsoft Bing Outperforms Five Other Generative Artificial Intelligence Chatbots in the Antwerp University Multiple Choice Medical License Exam. PLoS Digit. Health.

[54] Uppalapati V.K., Nag D.S. (2024). A Comparative Analysis of AI Models in Complex Medical Decision-Making Scenarios: Evaluating ChatGPT, Claude AI, Bard, and Perplexity. Cureus.

[55] Gravina A.G., Pellegrino R., Palladino G., Imperio G., Ventura A., Federico A. (2024). Charting New AI Education in Gastroenterology: Cross-Sectional Evaluation of ChatGPT and Perplexity AI in Medical Residency Exam. Dig. Liver Dis..

[56] Singer M.B., Fu J.J., Chow J., Teng C.C. (2024). Development and Evaluation of Aeyeconsult: A Novel Ophthalmology Chatbot Leveraging Verified Textbook Knowledge and GPT-4. J. Surg. Educ..

[57] Borji A., Mohammadian M. (2023). Battle of the Wordsmiths: Comparing ChatGPT, GPT-4, Claude, and Bard. SSRN Electron. J..

[58] Burnell R., Hao H., Conway A.R.A., Orallo J.H. (2023). Revealing the Structure of Language Model Capabilities. arXiv.

[59] Almazrouei E., Alobeidli H., Alshamsi A., Cappelli A., Cojocaru R., Debbah M., Goffinet É., Hesslow D., Launay J., Malartic Q. (2023). The Falcon Series of Open Language Models. arXiv.

[60] Bringing Grok to Everyone. https://x.ai/.

[61] Li Y., Li Z., Zhang K., Dan R., Jiang S., Zhang Y. (2023). ChatDoctor: A Medical Chat Model Fine-Tuned on a Large Language Model Meta-AI (LLaMA) Using Medical Domain Knowledge. Cureus.

[62] Cabezas D., Fonseca-Delgado R., Reyes-Chacón I., Vizcaino-Imacaña P., Morocho-Cayamcela M. (2024). Integrating a LLaMa-Based Chatbot with Augmented Retrieval Generation as a Complementary Educational Tool for High School and College Students. Proceedings of the 19th International Conference on Software Technologies.

[63] Llama the Open-Source AI Models You Can Fine-Tune, Distill and Deploy Anywhere. Choose from Our Collection of Models: Llama 3.1, Llama 3.2, Llama 3.3. https://www.llama.com/.

[64] Şahin M.F., Topkaç E.C., Doğan Ç., Şeramet S., Özcan R., Akgül M., Yazıcı C.M. (2024). Still Using Only ChatGPT? The Comparison of Five Different Artificial Intelligence Chatbots’ Answers to the Most Common Questions About Kidney Stones. J. Endourol..

[65] Sai S., Gaur A., Sai R., Chamola V., Guizani M., Rodrigues J.J.P.C. (2024). Generative AI for Transformative Healthcare: A Comprehensive Study of Emerging Models, Applications, Case Studies, and Limitations. IEEE Access.

[66] Sevgi M., Antaki F., Keane P.A. (2024). Medical Education with Large Language Models in Ophthalmology: Custom Instructions and Enhanced Retrieval Capabilities. Br. J. Ophthalmol..

[67] Sabaner M.C., Hashas A.S.K., Mutibayraktaroglu K.M., Yozgat Z., Klefter O.N., Subhi Y. (2024). The Performance of Artificial Intelligence-Based Large Language Models on Ophthalmology-Related Questions in Swedish Proficiency Test for Medicine: ChatGPT-4 Omni vs Gemini 1.5 Pro. AJO Int..

[68] Mihalache A., Huang R.S., Mikhail D., Popovic M.M., Shor R., Pereira A., Kwok J., Yan P., Wong D.T., Kertes P.J. (2024). Interpretation of Clinical Retinal Images Using an Artificial Intelligence Chatbot. Ophthalmol. Sci..

[69] Moshirfar M., Altaf A.W., Stoakes I.M., Tuttle J.J., Hoopes P.C. (2023). Artificial Intelligence in Ophthalmology: A Comparative Analysis of GPT-3.5, GPT-4, and Human Expertise in Answering StatPearls Questions. Cureus.

[70] Mihalache A., Huang R.S., Popovic M.M., Patil N.S., Pandya B.U., Shor R., Pereira A., Kwok J.M., Yan P., Wong D.T. (2024). Accuracy of an Artificial Intelligence Chatbot’s Interpretation of Clinical Ophthalmic Images. JAMA Ophthalmol..

[71] Huang X., Raja H., Madadi Y., Delsoz M., Poursoroush A., Kahook M.Y., Yousefi S. (2024). Predicting Glaucoma Before Onset Using a Large Language Model Chatbot. Am. J. Ophthalmol..

[72] Cascella M., Semeraro F., Montomoli J., Bellini V., Piazza O., Bignami E. (2024). The Breakthrough of Large Language Models Release for Medical Applications: 1-Year Timeline and Perspectives. J. Med. Syst..

[73] Yang Z., Wang D., Zhou F., Song D., Zhang Y., Jiang J., Kong K., Liu X., Qiao Y., Chang R.T. (2024). Understanding Natural Language: Potential Application of Large Language Models to Ophthalmology. Asia-Pac. J. Ophthalmol..

[74] Claude Is a Next Generation AI Assistant Built by Anthropic. https://claude.ai/.

[75] Hirosawa T., Harada Y., Tokumasu K., Shiraishi T., Suzuki T., Shimizu T. (2024). Comparative Analysis of Diagnostic Performance: Differential Diagnosis Lists by LLaMA3 Versus LLaMA2 for Case Reports. JMIR Form. Res..

[76] Balas M., Wong D.T., Arshinoff S.A. (2024). Artificial Intelligence, Adversarial Attacks, and Ocular Warfare. AJO Int..

[77] Maywood M.J., Parikh R., Deobhakta A., Begaj T. (2024). Performance Assessment of an Artificial Intelligence Chatbot in Clinical Vitreoretinal Scenarios. Retina.

[78] Carlà M.M., Gambini G., Baldascino A., Boselli F., Giannuzzi F., Margollicci F., Rizzo S. (2024). Large Language Models as Assistance for Glaucoma Surgical Cases: A ChatGPT vs. Google Gemini Comparison. Graefe’s Arch. Clin. Exp. Ophthalmol..

[79] Tan D.N.H., Tham Y.-C., Koh V., Loon S.C., Aquino M.C., Lun K., Cheng C.-Y., Ngiam K.Y., Tan M. (2024). Evaluating Chatbot Responses to Patient Questions in the Field of Glaucoma. Front. Med..

[80] Schumacher I., Bühler V.M.M., Jaggi D., Roth J. (2024). Artificial Intelligence Derived Large Language Model in Decision-Making Process in Uveitis. Int. J. Retin. Vitr..

[81] Balas M., Mandelcorn E.D., Yan P., Ing E.B., Crawford S.A., Arjmand P. (2024). ChatGPT and Retinal Disease: A Cross-Sectional Study on AI Comprehension of Clinical Guidelines. Can. J. Ophthalmol..

[82] Balas M., Janic A., Daigle P., Nijhawan N., Hussain A., Gill H., Lahaie G.L., Belliveau M.J., Crawford S.A., Arjmand P. (2024). Evaluating ChatGPT on Orbital and Oculofacial Disorders: Accuracy and Readability Insights. Ophthalmic Plast. Reconstr. Surg..

[83] Yalla G.R., Hyman N., Hock L.E., Zhang Q., Shukla A.G., Kolomeyer N.N. (2024). Performance of Artificial Intelligence Chatbots on Glaucoma Questions Adapted From Patient Brochures. Cureus.

[84] Yılmaz İ.E., Doğan L. (2024). Talking Technology: Exploring Chatbots as a Tool for Cataract Patient Education. Clin. Exp. Optom..

[85] Momenaei B., Wakabayashi T., Shahlaee A., Durrani A.F., Pandit S.A., Wang K., Mansour H.A., Abishek R.M., Xu D., Sridhar J. (2023). Appropriateness and Readability of ChatGPT-4-Generated Responses for Surgical Treatment of Retinal Diseases. Ophthalmol. Retin..

[86] Anguita R., Makuloluwa A., Hind J., Wickham L. (2024). Large Language Models in Vitreoretinal Surgery. Eye.

[87] Lyons R.J., Arepalli S.R., Fromal O., Choi J.D., Jain N. (2024). Artificial Intelligence Chatbot Performance in Triage of Ophthalmic Conditions. Can. J. Ophthalmol..

[88] Milad D., Antaki F., Milad J., Farah A., Khairy T., Mikhail D., Giguère C.-É., Touma S., Bernstein A., Szigiato A.-A. (2024). Assessing the Medical Reasoning Skills of GPT-4 in Complex Ophthalmology Cases. Br. J. Ophthalmol..

[89] Potapenko I., Boberg-Ans L.C., Stormly Hansen M., Klefter O.N., van Dijk E.H.C., Subhi Y. (2023). Artificial Intelligence-based Chatbot Patient Information on Common Retinal Diseases Using ChatGPT. Acta Ophthalmol..

[90] Rasmussen M.L.R., Larsen A.-C., Subhi Y., Potapenko I. (2023). Artificial Intelligence-Based ChatGPT Chatbot Responses for Patient and Parent Questions on Vernal Keratoconjunctivitis. Graefe’s Arch. Clin. Exp. Ophthalmol..

[91] Eriksen N.S., Al-Bakri M., Boysen K.B., Klefter O.N., Schmidt D.C., Reinwaldt K., Grauslund J., Holm L.M., Subhi Y. (2024). Generative Artificial Intelligence for Increasing Accessibility of Patient Information Videos in Ophthalmology. AJO Int..

[92] Taloni A., Borselli M., Scarsi V., Rossi C., Coco G., Scorcia V., Giannaccare G. (2023). Comparative Performance of Humans versus GPT-4.0 and GPT-3.5 in the Self-Assessment Program of American Academy of Ophthalmology. Sci. Rep..

[93] The International Committee of Medical Journal Editors Recommendations for the Conduct, Reporting, Editing, and Publication of Scholarly Work in Medical Journals. https://www.icmje.org/icmje-recommendations.pdf.

[94] Federspiel F., Mitchell R., Asokan A., Umana C., McCoy D. (2023). Threats by Artificial Intelligence to Human Health and Human Existence. BMJ Glob. Health.

[95] Antaki F., Milad D., Chia M.A., Giguère C.-É., Touma S., El-Khoury J., Keane P.A., Duval R. (2024). Capabilities of GPT-4 in Ophthalmology: An Analysis of Model Entropy and Progress towards Human-Level Medical Question Answering. Br. J. Ophthalmol..

[96] Fowler T., Pullen S., Birkett L. (2023). Performance of ChatGPT and Bard on the Official Part 1 FRCOphth Practice Questions. Br. J. Ophthalmol..

[97] Tao B.K.L., Hua N., Milkovich J., Micieli J.A. (2024). ChatGPT-3.5 and Bing Chat in Ophthalmology: An Updated Evaluation of Performance, Readability, and Informative Sources. Eye.

[98] Haddad F., Saade J.S. (2024). Performance of ChatGPT on Ophthalmology-Related Questions Across Various Examination Levels: Observational Study. JMIR Med. Educ..

[99] Cai L.Z., Shaheen A., Jin A., Fukui R., Yi J.S., Yannuzzi N., Alabiad C. (2023). Performance of Generative Large Language Models on Ophthalmology Board–Style Questions. Am. J. Ophthalmol..

[100] Antaki F., Touma S., Milad D., El-Khoury J., Duval R. (2023). Evaluating the Performance of ChatGPT in Ophthalmology. Ophthalmol. Sci..

[101] Mihalache A., Popovic M.M., Muni R.H. (2023). Performance of an Artificial Intelligence Chatbot in Ophthalmic Knowledge Assessment. JAMA Ophthalmol..

[102] Müller C., Mildenberger T. (2021). Facilitating Flexible Learning by Replacing Classroom Time with an Online Learning Environment: A Systematic Review of Blended Learning in Higher Education. Educ. Res. Rev..

[103] Al-Maroof R., Al-Qaysi N., Salloum S.A., Al-Emran M. (2022). Blended Learning Acceptance: A Systematic Review of Information Systems Models. Technol. Knowl. Learn..

[104] Černý M. (2023). Educational Psychology Aspects of Learning with Chatbots without Artificial Intelligence: Suggestions for Designers. Eur. J. Investig. Health Psychol. Educ..

[105] Moldt J.A., Festl-Wietek T., Madany Mamlouk A., Nieselt K., Fuhl W., Herrmann-Werner A. (2023). Chatbots for Future Docs: Exploring Medical Students’ Attitudes and Knowledge towards Artificial Intelligence and Medical Chatbots. Med. Educ. Online.

[106] Chakraborty C., Pal S., Bhattacharya M., Dash S., Lee S.S. (2023). Overview of Chatbots with Special Emphasis on Artificial Intelligence-Enabled ChatGPT in Medical Science. Front. Artif. Intell..

[107] Clark M., Bailey S. (2024). Chatbots in Health Care: Connecting Patients to Information. Can. J. Health Technol..

[108] Li J. (2023). Security Implications of Ai Chatbots in Health Care. J. Med. Internet Res..

[109] Lee P., Bubeck S., Petro J. (2023). Benefits, Limits, and Risks of GPT-4 as an AI Chatbot for Medicine. N. Engl. J. Med..

[110] Singh J., Sillerud B., Singh A. (2023). Artificial Intelligence, Chatbots and ChatGPT in Healthcare—Narrative Review of Historical Evolution, Current Application, and Change Management Approach to Increase Adoption. J. Med. Artif. Intell..

[111] Gonzalez K., Kanitz R., Briker R. (2024). “AI Can’t Steal My Soul”: In the Age of AI, the Human Touch Is Paramount for the Craft of Managing Change. J. Appl. Behav. Sci..

[112] Goumas G., Dardavesis T.I., Syrigos K., Syrigos N., Simou E. (2024). Chatbots in Cancer Applications, Advantages and Disadvantages: All That Glitters Is Not Gold. J. Pers. Med..

[113] Anguita R., Ferro Desideri L., Loewenstein A., Zinkernagel M. (2024). The Digital Age in Retinal Practice. Int. J. Retin. Vitr..

